# Microbial community compositions in the gastrointestinal tract of Chinese Mongolian sheep using Illumina MiSeq sequencing revealed high microbial diversity

**DOI:** 10.1186/s13568-017-0378-1

**Published:** 2017-04-04

**Authors:** Yan Zeng, Dong Zeng, Xueqin Ni, Hui Zhu, Ping Jian, Yi Zhou, Shuai Xu, Yicen Lin, Yang Li, Zhongqiong Yin, Kangcheng Pan, Bo Jing

**Affiliations:** 1 Animal Microecology Institute, College of Veterinary Medicine, Sichuan Agricultural University, Chengdu, 611130 Sichuan China; 2 Key Laboratory of Animal Disease and Human Health of Sichuan Province, Chengdu, 611130 Sichuan China

**Keywords:** Chinese Mongolian sheep, Gastrointestinal tract, Illumina MiSeq, Microbiota, Metabolic pathways

## Abstract

Chinese Mongolian sheep are an important ruminant raised for wool and meat production. However, little is known about the microbiota of the gastrointestinal tract (GIT) of Chinese Mongolian sheep. To increase our understanding of the microbial community composition in the GIT of Chinese Mongolian sheep, microbiota of five sheep is investigate for the first time using the Illumina MiSeq platform. High microbial diversity was obtained from the GIT, and the microbiota exhibited a higher biodiversity in the stomach and large intestine than in the small intestine. *Firmicutes* (44.62%), *Bacteroidetes* (38.49%), and *Proteobacteria* (4.11%) were the three most abundant phyla present in the GIT of the sheep. The present study also revealed the core genera of *Prevotella*, *Bacteroides*, *Ruminococcus*, *Oscillospira*, *Treponema*, and *Desulfovibrio* in the GIT. Phylogenetic Investigation of Communities by Reconstruction of Unobserved States indicated that the metabolic pathway related to carbohydrate metabolism was the richest in the sheep GIT. In addition, a series of metabolic pathways related to plant secondary metabolism was most abundant in the stomach and large intestine than in the small intestine. Overall, the present study provides insight into the microbial community composition in GIT of the Chinese Mongolian sheep which is highly diverse and needs to be studied further to exploit the complex interactions with the host.

**Electronic supplementary material:**

The online version of this article (doi:10.1186/s13568-017-0378-1) contains supplementary material, which is available to authorized users.

## Introduction

Microbiota of mammalian gastrointestinal tract (GIT) is a complex ecosystem constitute of diverse bacterial populations (Falony et al. 2016). The intra- and interpersonal variation in the composition of the human microbiome significantly complicates the analysis of microbiome data (Consortium HMP 2012; Taglialatela et al. 2009). The recently proposed concept of enterotypes or stool community types has overcome this difficulty (Arumugam et al. 2011; Koren et al. 2013). Gut microbiota assists in intestinal homeostasis and other aspects of the host, including intestinal immune response, digestion, physiology, and disease treatment (Donia et al. 2014; Koboziev et al. 2014). The microflora in the GIT of ruminants plays a critical role in fiber degradation (Nyonyo et al. 2014; Thoetkiattikul et al. 2013). Ruminants can efficiently digest dietary fiber and absorb nutrients because of their unique stomachs, including the rumen, reticulum, omasum, and abomasum, particularly the rumen (Morgavi et al. 2013). Due to the easiest sampling procedure feces and rumen of ruminants were most studied (Kittelmann et al. 2013; Lee et al. 2011). The microbiota in the stomach, small intestine, and large intestine of koalas, hoatzins, Brazilian Nelore steer, and mice has been recently explored through high-throughput next-generation sequencing (Barker et al. 2013; de Oliveira et al. 2013; Godoy-Vitorino et al. 2012; Gu et al. 2013).

Chinese Mongolian sheep are an important ruminant raised for wool and meat production; this animal is the source of one of the three most common varieties of coarse wool sheep in China (Zhang et al. 2008). In our previous study, we successfully characterized the cellulolytic bacterial communities along the GIT of Chinese Mongolian sheep through polymerase chain reaction-denaturing gradient gel electrophoresis (PCR-DGGE) and real-time PCR analyses (Zeng et al. 2015). Various bacteria thrive along the GIT, and these bacteria are more abundant in the stomach and large intestine than in the small intestine. We thus hypothesized that abundant gut microbiota information can be obtained from the GIT of Chinese Mongolian sheep through high-throughput next-generation sequencing. The samples in the Zeng et al. (2015) is identical to the ones in this study. Therefore, Illumina MiSeq platform was used to explore the bacteria diversity and composition in the GIT of Chinese Mongolian sheep.

## Materials and methods

### Animals and sampling

Samples were collected from five two-year-old male healthy Chinese Mongolian sheep (48.16 ± 1.48 kg body weight). These animals were reared and maintained in Gansu, China, in accordance with the standard livestock management practices. A diet of corn silage was provided in accordance with the agricultural industry standard of the People’s Republic of China (NYT816-2004). Animals were butchered in accordance with the approved by the Sichuan Agricultural University Committee on Ethics in the Care and Use of Laboratory Animals (Permit No. DKY-S20123517). Fresh samples (20 g) were collected from different segments of the GIT, namely, they were the stomach (rumen, reticulum, omasum, and abomasum), small intestine (duodenum, jejunum, and ileum), and large intestine (cecum, colon, and rectum). Finally, 50 samples were placed in sterile centrifuge tubes and immediately frozen in liquid nitrogen containers. The samples were then stored at −80 °C until further analysis. All samples were analyzed within a month.

### DNA extraction

Bacteria DNA was extracted from GIT samples (100 mg each) in accordance with the instructions of the EZNA Stool DNA Kit (Omega Bio-tek). Sterile zirconia beads were used to increase the extraction yield and the quality of the bacteria DNA (Yu and Morrison 2004). All procedures were performed on ice. The final elution volume was 200 μL, and DNA concentration was conducted on a Nano Drop spectrophotometer (Nano Drop Technologies, Wilmington, DE, USA). DNA samples were then stored frozen (−20 °C) until further analysis.

### 16S rRNA amplification and MiSeq sequencing

The V4 region of the 16S rRNA gene was amplified from genomic DNA using the following primers: 515F, 5′-GTGCCAGCMGCCGCGGTAA-3′; 806R, 5′-GGACTA CHVGGGTWTCTAAT-3′ (Caporaso et al. 2011). The reverse primer was barcoded with a unique 6 bp error-correcting to each sample. In brief, PCR was performed in triplicate in a 20 μL reaction mixture containing 10 ng of template DNA, 0.2 μM of each primer, 4 μL of 5× FastPfu Buffer, 0.4 μL of FastPfu Polymerase, and 2 μL of 2.5 mM dNTPs (MBI Fermentas, Waltham, MA, USA). The following thermal cycling conditions were used: 3 min of initial denaturation at 94 °C; 35 cycles of denaturation at 94 °C for 45 s, annealing at 50 °C for 60 s, and elongation at 72 °C for 90 s; and a last step at 72 °C for 10 min. The amplified products were evaluated by electrophoresis in 2% agarose gel and purified with the QIAquick Gel Extraction Kit (Qiagen, Dusseldorf, Germany). After purification, the samples were quantified using a Nano Drop spectrophotometer (Nano Drop Technologies, Wilmington, DE, USA). Sequencing was performed on an Illumina MiSeq 2 × 250 platform (Illumina, Inc. San Diego) at the Beijing Genomics Institute (Shanghai, China) in accordance with a previously described protocol (Caporaso et al. 2012).

### Data analysis

Bioinformatics analysis was performed on the website (http://www.mothur.org/wiki/MiSeq_SOP). The bacterial sequence reads were assembled using mothur v 1.32 (Schloss et al. 2009). To increase the analysis quality, the USEARCH software was used to remove chimeric sequences (Edgar et al. 2011). Operational Taxonomic Units (OTUs) were selected by using sequences with a 3% dissimilarity level. The microbial diversity structures (alpha and beta diversity) in different samples were analyzed using the QIIME software (Caporaso et al. 2010) with Python scripts. Alpha diversity was performed using the Shannon index, Chao 1, Observed species index and Simpson index. The taxonomy assignment of OTUs was investigated by comparing sequences to the Green-gene (http://rdp.cme.msu.edu/). Gene prediction was performed using PICRUSt 1.0.0 and Greengenes database v13.5 (Langille et al. 2013). The Venn diagrams were constructed on the basis of the relative abundance of bacteria on the level of genus. The R packages “Biom”, “Phyloseq”, and “Pheatmap” were used for data analysis and plotting (Mcdonald et al. 2012; Mcmurdie and Holmes 2013). The original sequencing data of raw reads were deposited in the sequence read archive of the National Center for Biotechnology (Aaccession Nos. PRJNA294127, SRR2242800, and SRS1048835).

## Results

### Metadata and sequencing

**Fig. 1:**
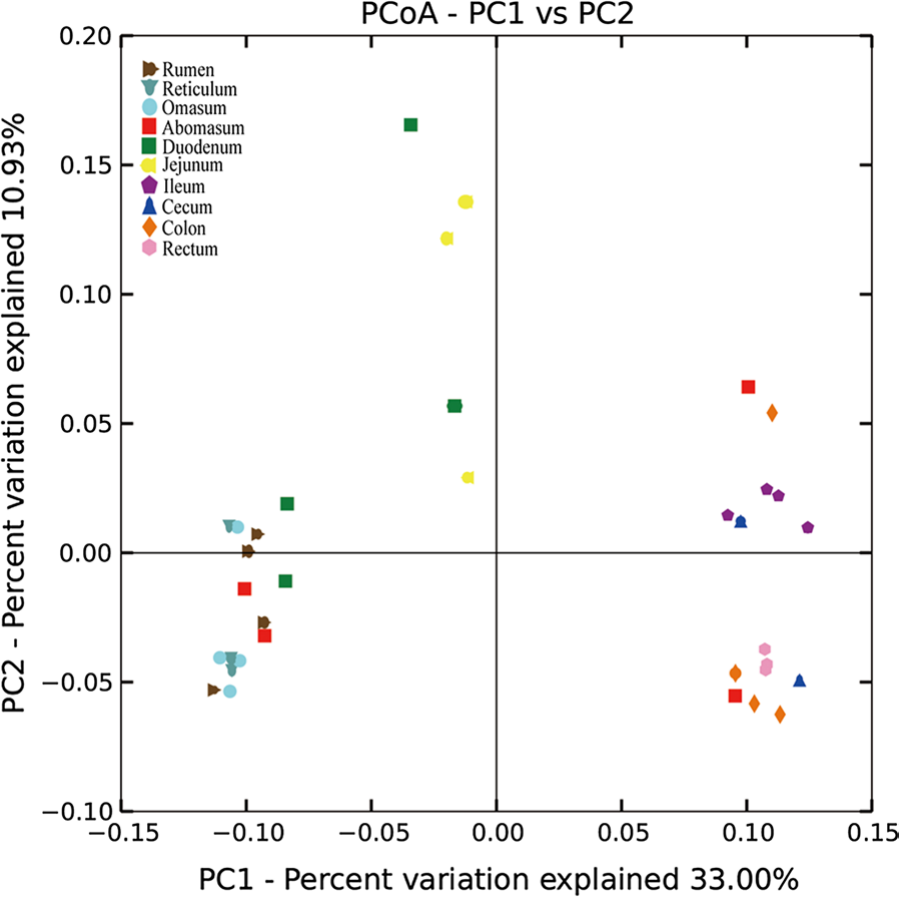
The PCoA analysis of the GIT samples (unweighted UniFrac metric). The *colored circles* represent the gut microbiota from the rumen, reticulum, omasum, abomasum, duodenum, jejunum, ileum, cecum, colon, and rectum, respectively

**Fig. 2:**
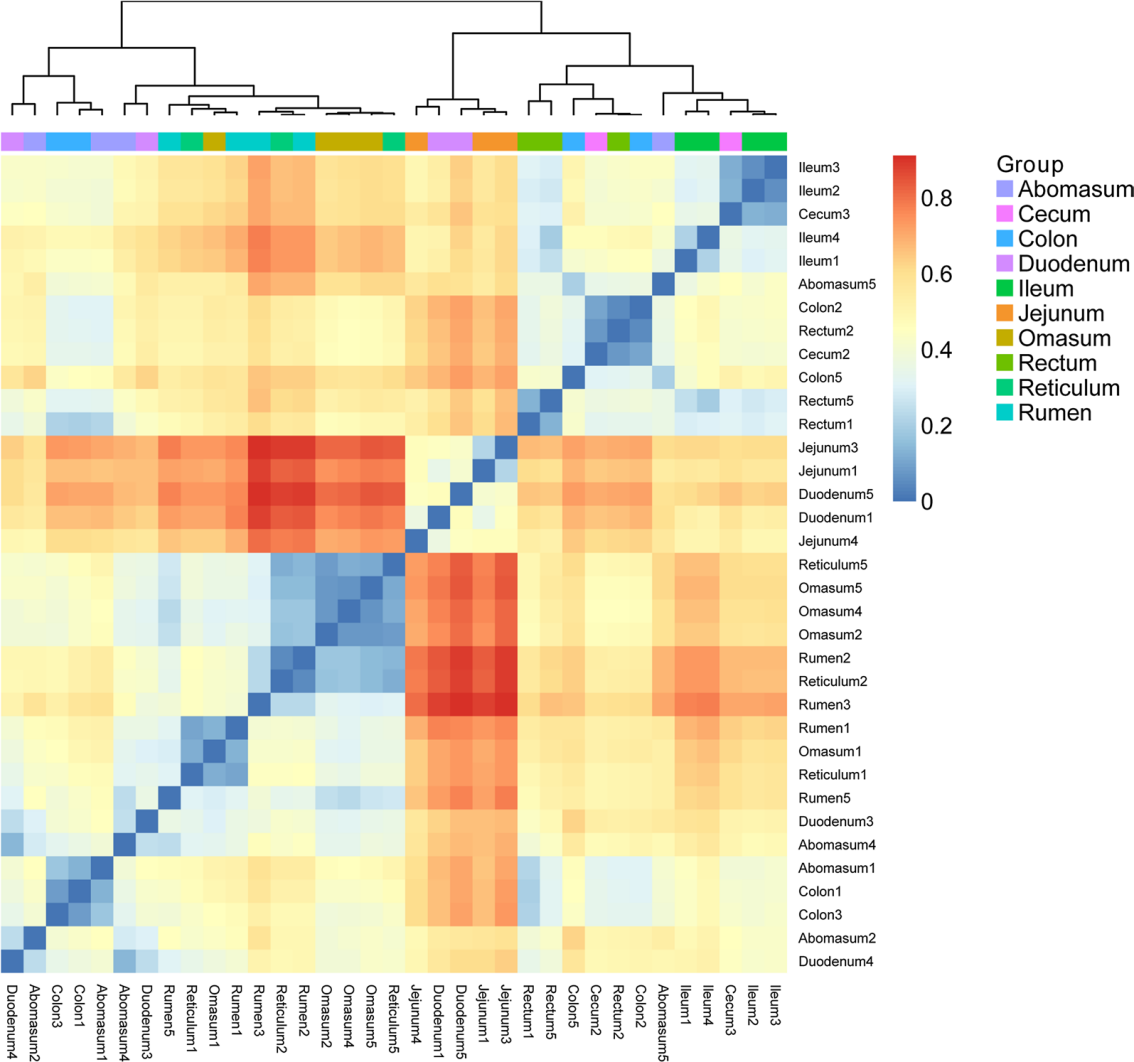
The heatmap of beta diversity of samples. The different color intensities represent the relative bacteria abundance in each sample. The number following the sample names stand for the sheep number. For example, reticulum 1, reticulum 2, reticulum 3, reticulum 4, and reticulum 5 stands for the reticulum samples from the 1st, 2nd, 3rd, 4th, and 5th sheep

Using the Illumian MiSeq platform of 16S rRNA gene amplicons, a total of 557,657 sequences with a median length of 252 base pairs (bp) (V4~533–786 bp) assigned to 16,252 OTUs were obtained from all samples (Additional file 1: Table S1). Each sample has 15,933 sequences and 464 OTUs on average (Additional file 1: Table S2). The bacterial communities in the GIT samples from sheep were divided into three clear groups, namely, stomach, small intestine, and large intestine, through principal coordinate analysis (PCoA) by using the UniFrac tool (Fig. 1). We evaluated the alpha (Chao, Ace, Shannon, and Simpson) and beta diversities of the bacterial community in the GIT (Additional file 1: Table S2; Fig. 2). The indices of alpha diversity were analyzed on the basis of OTUs. The bacterial diversity was higher in the stomach and large intestine than in the small intestine. The Chao, Ace, Shannon, and Simpson indices of bacterial communities ranged from 190 to 882, 200 to 863, 2.55 to 5.33, and 0.0111 to 0.1929, respectively. No significant difference was observed among the samples obtained from the same compartment in five individuals (*P* < 0.05). Meanwhile, the beta diversity showed a degree of diversity discrepancy in all samples.

According to the SILVA taxonomic database, all sequences of the samples were classified from phylum to species by using the QIIME program. In this study, the classifications of relative abundance of bacterial OTUs from phylum, class, order, family, genus, and species were shown with heatmaps (Additional file 1: Figure S1a–f). On the basis of the classifications (>1%), the segments of GIT from Chinese Mongolian sheep harbored bacteria from 11 phyla (e.g., *Firmicutes*, 44.62%; *Bacteroidetes*, 38.49%; *Proteobacteria*, 4.11%; *Spirochaetes*, 3.44%; and *Euryarchaeota*, 1.78%), 19 classes (e.g., *Clostridia*, 42.02%; *Bacteroidia*, 37.30%; *Spirochaetes*, 3.36%; Unclassified, 2.80%; and *Fibrobacteria*, 0.58%), 20 orders (e.g., *Clostridiales*, 42.01%; *Bacteroidales*, 37.30%; *Spirochaetales*, 3.26%; Unclassified, 2.90%; and *Methanobacteriales*, 1.83%), 38 families (e.g., *Ruminococcaceae*, 20.76%; *Prevotellaceae*, 16.60%; *Lachnospiraceae*, 8.37%; Unclassified, 6.88%; and *Bacteroidaceae*, 5.23%), 40 genera (e.g., Unknown, 20.76%; Unclassified, 19.92%; *Prevotella*, 15.56%; *Ruminococcus*, 6.35%; and *Treponema*, 3.26%), and 18 species (e.g., Unknown, 63.42%; Unclassified, 21.70%; *Prevotella ruminicola*, 5.45%; *Ruminococcus flavefaciens*, 3.63%; and *Ruminobacter albus*, 1.72%).

**Fig. 3:**
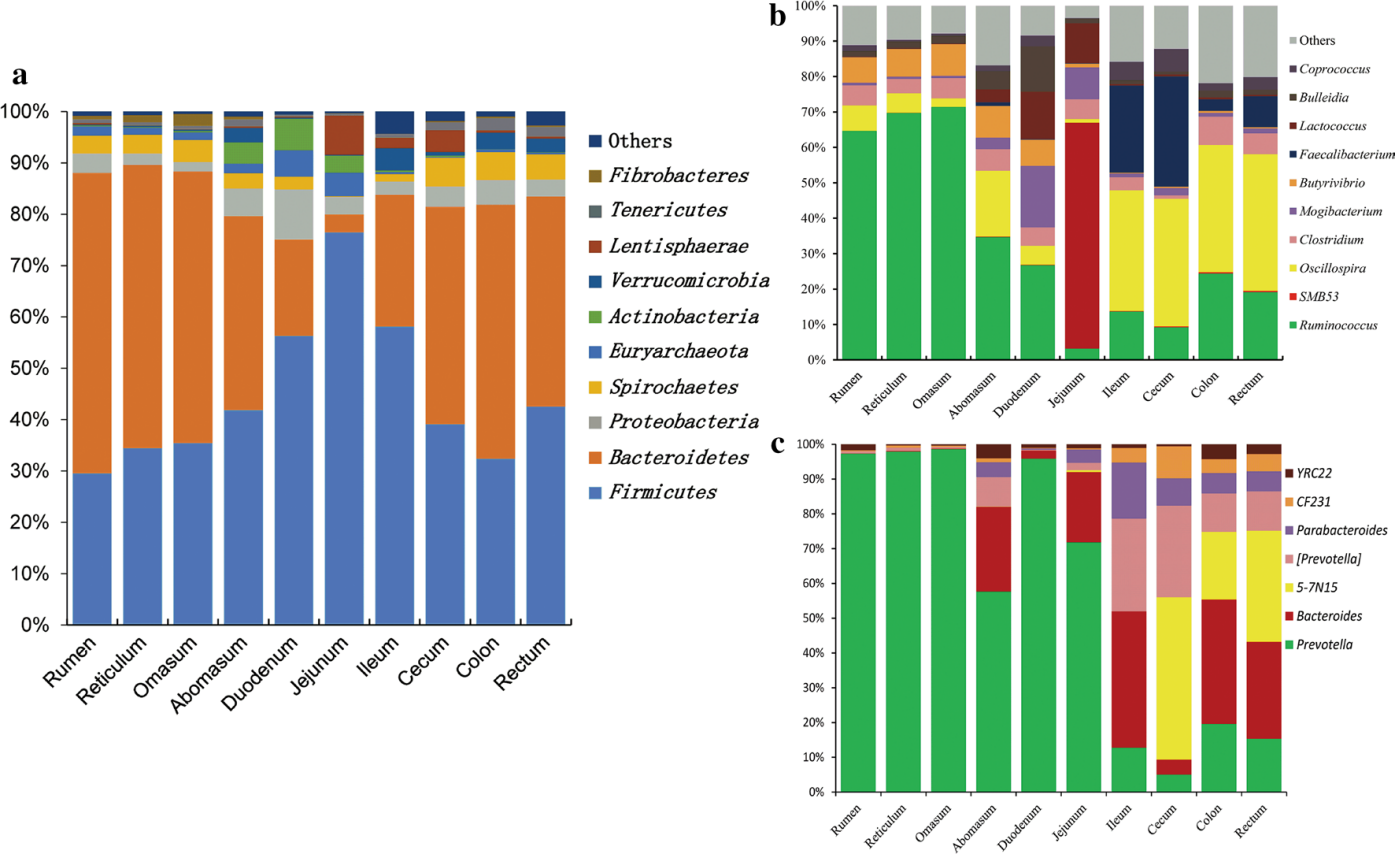
GIT microbiota at the phylum and genus level. Relative abundance of OTUs at the phylum level in individuals (**a**). Relative abundance of OTUs from *Firmicutes* (**b**) and *Bacteroidetes* (**c**) at the genus level in individuals. Only phyla or genera with greater than 1% representation are shown

The results shown in Fig. 3a describe the composition of the bacterial communities in the GIT at the phylum level. Among these phyla, *Firmicutes* and *Bacteroidetes* were the most predominant. The number of *Bacteroidetes* was higher in the stomach and large intestine than in the small intestine. Meanwhile, reverse results were obtained in *Firmicutes*. To analyze further the composition of the bacterial communities, we demonstrated the genera from *Firmicutes* and *Bacteroidetes* (Fig. 3b, c), respectively. The 10 most abundant genera from *Firmicutes* were *Ruminococcus*, *SMB53*, *Oscillospira*, *Clostridium*, *Mogibacterium*, *Butyrivibrio*, *Faecalibacterium*, *Lactococcus*, *Bulleidia*, and *Coprococcus*. The seven most abundant genera from *Bacteroidetes* were *Prevotella*, *Bacteroides*, *5*-*7N15*, [*Prevotella*]*, Parabacteroides*, *CF231*, and *YRC22*.

### Unique and shared bacterial genera in the sheep GIT

**Fig. 4:**
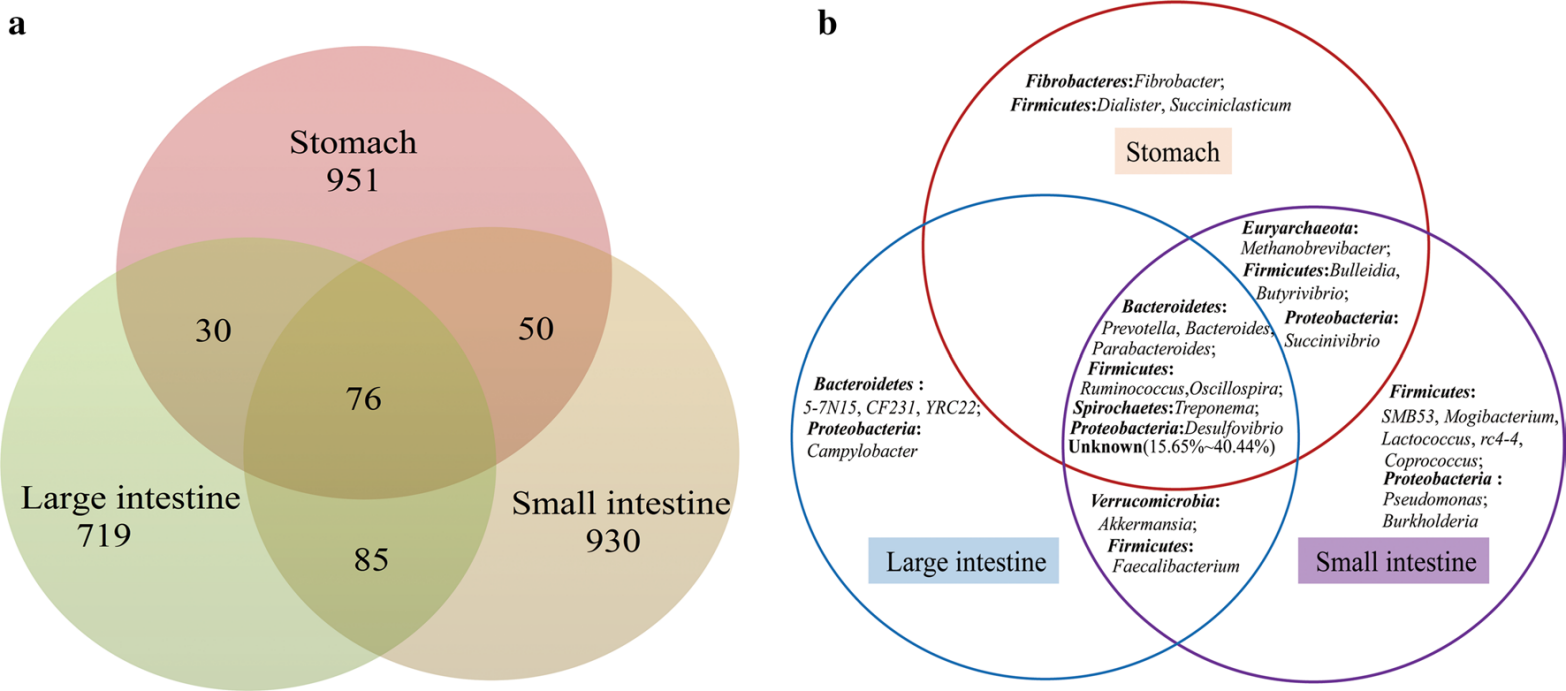
Venn diagrams of shared OTUs and bacterial genera. The shared OTUs between the stomach, small intestine, and large intestine microbiomes (**a**). The unique and shared bacterial genera (with the percentage of >1% colonized in segment) at the genus level in the sheep GIT (**b**)

We detected unique and shared bacterial genera along the GIT from sheep at the genus level by using our sequencing data. The genera with an average abundance >0.1% were analyzed using Venn diagrams (Fig. 4a). Surprisingly, a large amount of unknown genera (15.65–40.44%) was discovered in the GIT from Chinese Mongolian sheep (Fig. 4b). A total of 27 genera were observed, and only 25.93% of them belonged to the shared bacterial genera, including three genera (*Prevotella*, *Bacteroides*, and *Parabacteroides*) from Bacteroidetes, two genera (*Ruminococcus* and *Oscillospira*) from *Firmicutes*, one genus (*Treponema*) from *Spirochaetes*, and one genus (*Desulfovibrio*) from *Proteobacteria*. We found four unique bacterial genera (*Methanobrevibacter*, *Bulleidia*, *Butyrivibrio*, and *Succinivibrio*) between the stomach and small intestine. In addition, two unique bacterial genera (*Akkermansia* and *Faecalibacterium*) were observed between the small intestine and large intestine. However, we did not find unique bacterial genera between the stomach and large intestine.

Every segment from the GIT of sheep harbors a complicated ecological bacterial community. Additional file 1: Figure S2a and Table S3 shows that *Oscillospira* was observed only in the rumen, reticulum, and abomasum. *Succinivbrio* was observed only in the rumen and abomasum, and *Fibrobacter* was discovered only in the reticulum and abomasum. In the small intestine (Additional file 1: Figure S2b, Table S4), *Prevotella* and *Ruminococcus* were shared only by the duodenum, jejunum, and ileum. *Methanobrevibacter*, *Lactococcus*, *Mogibacterium*, and *Pseudomonas* were discovered only in the duodenum and jejunum. *Treponema* and *Oscillospira* were discovered only in the duodenum and ileum. As to the large intestine (Additional file 1: Figure S2c, Table S5), eight bacterial genera, namely, *Prevotella*, 5-7N15, *Bacteroides*, CF231, *Parabacteroides*, *Treponema*, *Oscillospira*, and *Ruminococcus*, were shared with the cecum, colon, and rectum. *Faecalibacterium* was observed only in the cecum and rectum, and *Campylobacter* was discovered only in the rectum.

### Bacterial function prediction in the GIT of sheep

**Fig. 5:**
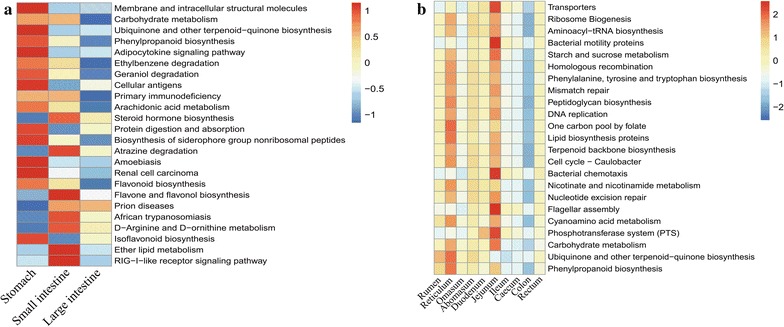
Predicted function of the gut micorbiota in the sheep of GIT. KEGG pathways were shown in two heatmaps. The bootstrap Mann–Whitney u-test was used to detect the gene distribution with cutoffs of P < 0.05, FDR <0.2, Mean counts >10,000 (**a**) and P < 0.01, FDR <0.1, Mean counts >10 (**b**)

Phylogenetic Investigation of Communities by Reconstruction of Unobserved States (PICRUSt) was used to predict the functional composition of the gut microbiota genomes in Chinese Mongolian sheep. The functional profiles are shown in Fig. 5. A total of 24 Kyoto Encyclopedia of Genes and Genomes (KEGG) pathways were found abundant in the stomach, small intestine, and large intestine (Fig. 5a, *P* < 0.01). Among these 24 KEGG pathways, eight (“Carbohydrate metabolism”, “Peptidoglycan biosynthesis”, “Ethylbenzene degradation”, “Geraniol degradation”, “Primary immunodeficiency”, “Arachidonic acid metabolism”, “Biosynthesis of siderophore group nonribosomal peptides”, and “Flagellar assembly”) primarily related to carbohydrate metabolism and bacterial flagellar assembly were more abundant in the stomach and small intestine than in the large intestine; three (“Membrane and intracellular structural molecules”, “Ubiquinone and other terpenoid-quinone biosynthesis”, and “Adipocytokine signaling pathway”) primarily related to the metabolism of cofactors and vitamins were significantly abundant only in the stomach; and two (“Ether lipid metabolism” and “RIG-I-like receptor signaling pathway”) were significantly abundant only in the small intestine. Careful analysis of each segment showed that 23 KEGG pathways were significantly more abundant in the rumen, reticulum, omasum, abomasum, duodenum, jejunum, ileum, cecum, colon, and rectum (Fig. 5b, *P* < 0.05). Three KEGG pathways (“One carbon pool by folate”, “Nicotinate and nicotinamide metabolism”, and “Ubiquinone and other terpenoid-quinone biosynthesis”) were significantly abundant only in the reticulum. However, five KEGG pathways (“Transporters”, “Bacterial motility proteins”, “Bacterial chemotaxis”, “Flagellar assembly”, and “Phosphotransferase system”) were significantly abundant only in the jejunum.

## Discussion

This study aimed to provide new insights into the diverse symbiotic bacterial communities along the GIT of Chinese Mongolian sheep. The gut microbiota co-developed with the host from birth is involved in the regulation of mammal’s immune function, digestion, physiology, and disease treatment (Koboziev et al. 2014). However, insufficient information is available about the microbial flora along the GIT of ruminants. In this study, we successfully characterized the microbiota in all segments of the GIT for the first time by using Illumina MiSeq. A remarkable microbiota composition was obtained from the GIT, and the microbiota showed a higher biodiversity in the stomach and large intestine than in the small intestine (Fig. 3; Additional file 1: Table S2). In this study, the microbiota varied along the GIT and was similar in the same segment of individual animals, which is in agreement with a previous finding (Koren et al. 2013). A recent study has reported that the foregut and hindgut of hoatzin and cow possess a relatively similar microbiota composition regardless of host species (Godoy-Vitorino et al. 2012). A similar result was obtained from our study; samples from adjacent parts of the GIT were clustered together (Fig. 1). The recently proposed concept of enterotypes and stool community types has overcome the difficulty in analyzing microbiome data because of intra- and interpersonal variation (Armstrong and Smithard 1979; Holmes et al. 2012; Koren et al. 2013; Turnbaugh et al. 2007).

In the present study, *Firmicutes*, *Bacteroidetes*, and *Proteobacteria* were predominantly abundant in all samples on average. These findings paralleled those of other studied on the microbiota in the GIT of ruminants (Cunha et al. 2011; Li et al. 2012b). Interestingly, the number of *Bacteroidetes* was higher in the stomach and large intestine than in the small intestine. Consistently, *Bacteroidetes* was found predominantly in the rumen, reticulum, and omasum of bovine (Peng et al. 2015), whereas reverse results were obtained in *Firmicutes*. *Bacteroidetes* aids in the digestion of complex carbohydrates (Spence et al. 2006), and *Firmicutes* is the dominant species in the GIT of ruminants and mainly consists of diverse fibrolytic and cellulolytic bacterial genera (Evans et al. 2011). In the present study, *Firmicutes* was more abundant in the small intestine than in the stomach and large intestine. This result is consistent with the findings in Brazilian Nelore steer (de Oliveira et al. 2013). However, reverse results were obtained in our previous study using real-time PCR (Zeng et al. 2015). This discrepancy is mainly attributed to the different primers used in real-time PCR and Illumina MiSeq. *Proteobacteria* comprises a large amount of bacteria that can catabolize feedstuff components (Evans et al. 2011), including corn and grass (Callaway et al. 2010). In the present study, *Proteobacteria* were predominantly abundant in the duodenum. However, another study on the South American folivorous hoatzin found that the number of *Proteobacteria* is lower in the foregut than in the hindgut (Godoy-Vitorino et al. 2012). In addition, *Fibrobacteres* is predominantly abundant in the omasum and the reticulum. These findings are consistent with our previous study (Zeng et al. 2015). In a previous study, the number of dominant fibrolytic bacteria, including *Ruminococcus albus*, *Fibrobacter succinogenes*, and *Ruminococcus flavefaciens*, is consistently higher in the stomach than in the large and small intestine (Zeng et al. 2015).

The results of 454 pyrosequencing showed that *Actinobacteria*, *Proteobacteria*, *Firmicutes*, and *Bacteroidetes* are predominantly abundant in all fecal samples of mammals, including 6 pigs, 14 healthy adult humans, 6 cows, 6 chickens, and 6 geese (Lee et al. 2011). In the present study, the genera *Prevotella*, *Bacteroides*, *Ruminococcus*, *Oscillospira*, *Treponema*, and *Desulfovibrio* were found in all samples. These genera belong to *Bacteroidetes*, *Firmicutes*, and *Proteobacteria*. *Prevotella* aids in the utilization of feed proteins in the rumen of ruminants (Xu and Gordon 2003) and can increase in abundance if the animal is fed a grain-based diet (Li et al. 2012a). *Prevotella* are sometimes believed to work in conjunction with the cellulolytic species *Fibrobacter succinogenes* in utilizing hemicellulose (Osborne and Dehority 1989). *Ruminococcus* plays a critical role in the digestion and metabolism of dietary fiber in ruminants (Han et al. 2015). Previous studies reported that *Treponema* is a genus of the primary bacterial community in the rumen; this genus reportedly disintegrates plant polysaccharides from ingested food (Avguštin et al. 1997; Bekele et al. 2011). Meanwhile, *Desulfovibrio* plays a significant role in the sulfate reduction of rumen and is more abundant in developing rumen than in mature rumen (Wu et al. 2012). Importantly, *Mogibacterium*, *Lactococcus*, *Pseudomonas*, and *Burkholderia* are abundant in the small intestine. *Mogibacterium* is a group of Gram-positive anaerobic bacteria that predominates the rumen of goats (Patel et al. 2011). The relative abundance of *Mogibacterium* could increase with high-grain feeding (Liu et al. 2015). *Coprococus*, which is abundant in the ileum, is an *Enterococcus* that can digest xylanolytic (Valdez-Vazquez et al. 2015). Our study provides evidence that the ileum may be another important segment of the GIT for dietary fiber. This observation agrees with the previous finding that *Coprococus* is a ubiquitous genus in Nelore GIT (de Oliveira et al. 2013). Surprisingly, *Campylobacter* species are abundant in the large intestine, especially in the cecum. Although this genus is usually known to comprise pathogenic bacteria, some species isolated from cattle and starlings show a high resistance to multiple antimicrobial drugs, including *ciprofloxacin*, *gentamicin*, and *erythromycin* (Sanad et al. 2013). Finally, *Fibrobacter*, *Dialister*, and *Succiniclasticum* were found more abundant in the stomach than in the small and large intestine. As reported, *Succiniclasticum* represents the majority of the sequence tags of the family *Veillonellaceae*, which belongs to the class *Clostridia* from *Firmicutes* in the three stomachs of bovine (Peng et al. 2015).

Microbiota function prediction revealed that most of the metabolic pathways in the GIT are related to carbohydrate metabolism. This finding is consistent with the observation that *Firmicutes* and *Bacteroidetes* are predominantly abundant in the GIT. Importantly, *Bacteroidetes* and *Firmicutes* are the dominant species aiding the digestion of complex carbohydrates in the GIT of ruminants (Evans et al. 2011; Spence et al. 2006). In the present study, higher diversity was detected in the stomach and large intestine than in the small intestine. Similarly, a previous research detected that the microbial fermentation and absorption of indigestible dietary substrates primarily occur in the rumen and colon and not in the small intestine (Abbeele et al. 2011). Another research reported that the rumen and colon of the North American moose are distinct environments (Ishaq and Wright 2012). In general, feed and fodder are first ingested and absorbed in the stomach of ruminants, the rest are ingested in the small and large intestine. In addition, the rumen is the most important segment of nutrient utilization (Kebreab et al. 2009), which primarily involves protein metabolism and plant secondary metabolism. Nevertheless, main nutrients, particularly proteins, are absorbed in the small intestine (Klieve 2005). Importantly, the rapid uptake and conversion of simple carbohydrates help maintain the micro-ecological balance of the small intestine (Zoetendal et al. 2012). In addition, indigested feed including some cellulose and starch can be completely but slowly assimilated in the large intestine (Armstrong and Smithard 1979). In the present study, we detected that some metabolic pathways related to microbial decomposition were significantly more abundant in the stomach than in the small intestine and large intestine. In addition, the metabolic pathways related to microbial synthesis (“Transporters”, “Ribosome Biogenesis”, and “Aminoacyl-tRNA biosynthesis”) were abundant in the reticulum. The reticulum is the second stomach along the GIT of ruminants and is conducive to the uniformity in the rumen fluid microbiome through the churning action with rumen (Braun 2009). A recent study has analyzed the bacterial composition of the rumen, reticulum, omasum, and abomasum of bovine for the first time by using a metagenomic approach (Peng et al. 2015). The primary composition of the microbiome was determined in the rumen, reticulum, and omasum. In addition, the metabolic pathways related to lipid metabolism and pattern-recognition receptors were significantly more abundant in the small intestine than in the stomach and large intestine. In general, lipid metabolism is primarily determined in the small intestine. Genes involved in the lipid metabolism are expressed in response to changes in the barrier lipids of the skin of sheep (Ovis aries) for their more significant role of volatile fatty acids (Jiang et al. 2014). We also detected that the metabolic pathways related to the motility of bacterial proteins and the chemotaxis of bacteria are significantly more abundant in the jejunum in other segments of the GIT. A previous in vitro study showed that the intestinal contents from the jejunum can digest cellulose and neutral detergent fiber (Jiao et al. 2013).

In the present study, according to the classifications (>1%) of relative abundance of bacterial OTUs from phylum, class, order, family, genus, and species, bacteria from 2.80% phyla, 2.90% class, 2.90% order, 19.92% family, 6.88% genera, and 63.42% species were unknown. Although we obtained a huge number of microbiota members (>93.12%) along the GIT of sheep on the level of genus, a large number of microbiota members (>63.42%) cannot be classified or remain unknown on the level of species. Recently, a metagenomic data analysis of the human gut has shown extensive strain-level variation across species, and differences in gene copy number affect specific adaptive functions (Greenblum et al. 2015). Upon the completion of the 1000 Genomes Project, scientists have proposed an interdisciplinary Unified Microbiome Initiative to discover and advance tools for understanding and harnessing the capabilities of various ecosystems of microbial communities, such as the human gut and marine ecosystems, to improve human health, agriculture, bio-energy, and the environment (Alivisatos et al. 2015; Consortium et al. 2015). Meanwhile, a similar study for animals must also be conducted in the future. Therefore, further analyses through metagenomics, metabolomics, and transcriptomics are needed to identify completely the microbiota along the GIT of Chinese Mongolian sheep.

In summary, we successfully for the first time characterized the bacterial taxa and metabolic pathways in all intestinal segments of Chinese Mongolian sheep by using Illumina MiSeq. Nevertheless, the obtained functional profiles are merely a prediction; detailed analyses are still needed to elucidate this aspect. Further studies are warranted to determine the contributions of the bacterial taxa and metabolic pathways to the health, development, and physiology of Chinese Mongolian sheep.

## Additional file

**Additional file 1.** Additional tables and figures.

## Abbreviations

GIT: gastrointestinal tract
PCR-DGGE: polymerase chain reaction-denaturing gradient gel electrophoresis
Real-time PCR: real-time polymerase chain reaction
OTUs: operational taxonomic units
QIIME: quantitative insights into microbial ecology
PCoA: principal coordinate analysis
