# Supplementary material for: Microbial community compositions in the gastrointestinal tract of Chinese Mongolian sheep using Illumina MiSeq sequencing revealed high microbial diversity
Source: AMB Express. 2017 Apr 4;7:75. doi: 10.1186/s13568-017-0378-1 (PMC5380569; doi:10.1186/s13568-017-0378-1)

**Journal Name: AMB Express**

**T****itle: Microbial community compositions in the gastrointestinal tract of Chinese Mongolian sheep using Illumina MiSeq sequencing revealed high microbial diversity**

**Authors:** Yan Zeng^1,2†^, Dong Zeng^1,2†^, Xueqin Ni^1,2*^, Hui Zhu^1,2^, Ping Jian^1,2^, Yi Zhou^1,2^, Shuai Xu^1,2^, Yicen Lin^1,2^, Yang Li^1,2^, Zhongqiong Yin^2^, Kangcheng Pan^1,2^ and Bo Jing^1,2^

^1^Animal Microecology Institute, College of Veterinary Medicine, Sichuan Agricultural University, Chengdu 611130, Sichuan, China.

^2^Key Laboratory of Animal Disease and Human Health of Sichuan Province, Chengdu 611130, Sichuan, China.

†Yan Zeng and Dong Zeng contributed equally to this work

**^*^Correspondence:** Dr. Xueqin Ni, [xueqinni@foxmail.com](mailto:xueqinni@foxmail.com)

Supplementary Table S1. OTU table summary ^a^

| **Sheep ID** | **Sample ID** | **Sequence counts** | **Observations (OTU number)** |
| --- | --- | --- | --- |
| Sheep 1 | Rumen 1 | 11,763 | 2,111 |
| Sheep 2 | Rumen 2 | 13,189 | 2,597 |
| Sheep 3 | Rumen 3 | 17,549 | 3,101 |
| Sheep 5 | Rumen 5 | 16,230 | 3,418 |
| Sheep 1 | Reticulum 1 | 25,705 | 4,374 |
| Sheep 2 | Reticulum 2 | 18,772 | 3,348 |
| Sheep 5 | Reticulum 5 | 21,068 | 4,125 |
| Sheep 1 | Omasum 1 | 13,795 | 2,113 |
| Sheep 2 | Omasum 2 | 15,038 | 3,326 |
| Sheep 4 | Omasum 4 | 13,840 | 2,817 |
| Sheep 5 | Omasum 5 | 12,282 | 2,701 |
| Sheep 1 | Abomasum 1 | 20,405 | 4,659 |
| Sheep 2 | Abomasum 2 | 16,706 | 1,829 |
| Sheep 4 | Abomasum 4 | 18,670 | 3,410 |
| Sheep 5 | Abomasum 5 | 18,629 | 3,597 |
| Sheep 1 | Duodenum 1 | 5,360 | 194 |
| Sheep 3 | Duodenum 3 | 9,023 | 1,705 |
| Sheep 4 | Duodenum 4 | 14,315 | 2,908 |
| Sheep 5 | Duodenum 5 | 20,722 | 1,862 |
| Sheep 1 | Jejunum 1 | 13,772 | 1,007 |
| Sheep 3 | Jejunum 3 | 15,043 | 1,184 |
| Sheep 4 | Jejunum 4 | 17,846 | 1,204 |
| Sheep 1 | Ileum 1 | 34,838 | 8,313 |
| Sheep 2 | Ileum 2 | 13,019 | 3,042 |
| Sheep 3 | Ileum 3 | 11,748 | 2,037 |
| Sheep 4 | Ileum 4 | 19,822 | 5,402 |
| Sheep 2 | Cecum 2 | 13,278 | 3,662 |
| Sheep 3 | Cecum 3 | 17,293 | 3,891 |
| Sheep 1 | Colon 1 | 16,216 | 3,708 |
| Sheep 2 | Colon 2 | 8,618 | 2,280 |
| Sheep 3 | Colon 3 | 6,329 | 1,776 |
| Sheep 5 | Colon 5 | 13,825 | 2,595 |
| Sheep 1 | Rectum 1 | 16,963 | 4,730 |
| Sheep 2 | Rectum 2 | 15,626 | 4,120 |
| Sheep 5 | Rectum 5 | 20,360 | 6,082 |

^a^ Due to a technical difficulty, the DNA from the samples, including rumen 4, reticulum 3, reticulum 3, omasum 3, abomasum 3, duodenum 2, jejunum 2, jejunum 5, ileum 5, cecum 1, cecum 4, cecum 5, colon 4, rectum 3, and rectum 4 were unexpectedly degraded during the sample processing and was not further analyzed in this study.

Supplementary Table S2. Summary of the Illumina MiSeq sequencing results^a^

| **Samples** | **Reads** | **OTU** | **Chao 1** | **Ace** | **Shannon** | **Simpson** |
| --- | --- | --- | --- | --- | --- | --- |
| Rumen | 11,763-17,549(16,230) | 332-403(370) | 430-548(497) | 442-532(489) | 3.60-4.28(3.96) | 0.0332-0.0948(0.0573) |
| Reticulum | 18,772-25,705(21,848) | 388-450(429) | 527-630(563) | 542-748(638) | 3.60-4.37(3.93) | 0.0111-0.0966(0.0661) |
| Omasum | 12,282-15,038(13,739) | 353-382(372) | 509-571(520) | 580-668(582) | 3.93-4.32(4.09) | 0.0456-0.0563(0.0467) |
| Abomasum | 16,706-20,405(18,603) | 469-600(513) | 597-701(635) | 592-701(627) | 4.31-4.82(4.53) | 0.0224-0.0410(0.0289) |
| Duodenum | 5,360-20,722(12,355) | 157-641(407) | 190-830(556) | 200-826(578) | 2.55-4.74(3.94) | 0.0164-0.2728(0.0905) |
| Jejunum | 13,772-17,846(15,554) | 298-694(453) | 525-852(645) | 698-860(754) | 2.56-3.61(3.01) | 0.1694-0.1929(0.1775) |
| Ileum | 11,748-34,838(19,857) | 452-749(559) | 536-882(668) | 544-863(661) | 4.71-5.09(4.91) | 0.0161-0.0222(0.0192) |
| Cecum | 12,278-17,293(15,286) | 490-512(501) | 609-611(610) | 332-403(611) | 4.55-4.57(4.56) | 0.0311-0.0246(0.0278) |
| Colon | 6,329-16,216(11,247) | 393-529(449) | 566-723(613) | 549-665(612) | 3.68-4.67(4.34) | 0.0226-0.1088(0.0474) |
| Rectum | 15,626-20,360(17,650) | 549-702(643) | 618-842(766) | 628-833(757) | 4.55-5.33(5.06) | 0.0111-0.0324(0.0179) |

^a^ The alpha diversity were calculated with 3％ distance cut-off.

Supplementary Table S3. Summary of the listed genera data in the stomach

| **Rumen** | | | |  | | **Reticulum** | | | | | |  | | | **Abomasum** | | | | |  | | **Omasum** | | | |
| --- | --- | --- | --- | --- | --- | --- | --- | --- | --- | --- | --- | --- | --- | --- | --- | --- | --- | --- | --- | --- | --- | --- | --- | --- | --- |
| Genera | | Mean | |  | | | Genera | Mean | | | |  | | | | Genera | Mean | |  | | | Genera | Mean | | |
| *Prevotella* | | | 0.4398 |  | *Prevotella* | | | | 0.4198 | | |  | *Prevotella* | | | | 0.3814 | |  | | Unknown | | | | 0.3139 |
| Unknown | | | 0.1817 |  | *Ruminococcus* | | | | 0.2003 | | |  | Unknown | | | | 0.2043 | |  | | *Prevotella* | | | | 0.1543 |
| *Ruminococcus* | | | 0.1500 |  | Unknown | | | | 0.1565 | | |  | *Ruminococcus* | | | | 0.1984 | |  | | *Bacteroides* | | | | 0.0742 |
| *Treponema* | | | 0.0438 |  | *Treponema* | | | | 0.0462 | | |  | *Treponema* | | | | 0.0533 | |  | | *Ruminococcus* | | | | 0.0694 |
| *Succinivibrio* | | | 0.0271 |  | *Butyrivibrio* | | | | 0.0265 | | |  | *Fibrobacter* | | | | 0.0278 | |  | | *Treponema* | | | | 0.0433 |
| *Methanobrevibacter* | | | 0.0270 |  | *Methanobrevibacter* | | | | 0.0204 | | |  | *Butyrivibrio* | | | | 0.0273 | |  | | *Desulfovibrio* | | | | 0.0405 |
| *Butyrivibrio* | | | 0.0188 |  | *Oscillospira* | | | | 0.0191 | | |  | *Methanobrevibacter* | | | | 0.0203 | |  | | *Oscillospira* | | | | 0.0402 |
| *Oscillospira* | | | 0.0182 |  | *Fibrobacter* | | | | 0.0159 | | |  |  | | | |  | |  | | *Methanobrevibacter* | | | | 0.0322 |
|  |  | | |  | *Dialister* | | | | 0.0123 | | |  |  | | | |  | |  | | *Butyrivibrio* | | | | 0.0174 |
|  | |  | |  | *Succiniclasticum* | | | | 0.0103 | | |  |  | | | |  | |  | | *Succinivibrio* | | | | 0.0157 |
|  | |  | |  | |  | | | |  |  | | |  | | | |  |  | | *Parabacteroides* | | | 0.0131 | |
|  | |  | |  | |  | | | |  |  | | |  | | | |  |  | | *Bulleidia* | | | 0.0107 | |

Supplementary Table S4. Summary of the listed genera data in the small intestine

| **Duodenum** | |  | **Ileum** | |  | **Jejunum** | |
| --- | --- | --- | --- | --- | --- | --- | --- |
| Genera | Mean |  | Genera | Mean |  | Genera | Mean |
| Unknown | 0.2921 |  | Unknown | 0.3808 |  | Unknown | 0.4044 |
| *Prevotella* | 0.1308 |  | *SMB53* | 0.3267 |  | *Bacteroides* | 0.0927 |
| *Ruminococcus* | 0.0757 |  | *Methanobrevibacter* | 0.0595 |  | *Oscillospira* | 0.0758 |
| *Methanobrevibacter* | 0.0735 |  | *Lactococcus* | 0.0523 |  | *Akkermansia* | 0.0639 |
| *Mogibacterium* | 0.0557 |  | *Mogibacterium* | 0.0412 |  | *Faecalibacterium* | 0.0533 |
| *Lactococcus* | 0.0488 |  | *Prevotella* | 0.0155 |  | *Parabacteroides* | 0.0359 |
| *Pseudomonas* | 0.0417 |  | *Ruminococcus* | 0.0153 |  | *Ruminococcus* | 0.0300 |
| *Bulleidia* | 0.0395 |  | *Pseudomonas* | 0.0115 |  | *Prevotella* | 0.0872 |
| *Treponema* | 0.0327 |  |  |  |  | *Treponema* | 0.0215 |
| *Succinivibrio* | 0.0239 |  |  |  |  | *rc4-4* | 0.0125 |
| *Butyrivibrio* | 0.0222 |  |  |  |  | *Coprococcus* | 0.0113 |
| *Oscillospira* | 0.0150 |  |  |  |  | *Desulfovibrio* | 0.0100 |
| *Burkholderia* | 0.0100 |  |  |  |  |  |  |

Supplementary Table S5. Summary of the listed genera data in the large intestine

| **Cecum** | |  | **Rectum** | |  | **Colon** | |
| --- | --- | --- | --- | --- | --- | --- | --- |
| Genera | Mean |  | Genera | Mean |  | Genera | Mean |
| Unknown | 0.3298 |  | Unknown | 0.2977 |  | Unknown | 0.3583 |
| *5-7N15* | 0.1831 |  | *Bacteroides* | 0.1532 |  | *5-7N15* | 0.1160 |
| *Prevotella* | 0.1738 |  | *5-7N15* | 0.0836 |  | *Bacteroides* | 0.1063 |
| *Treponema* | 0.0771 |  | *Prevotella* | 0.1308 |  | *Treponema* | 0.0698 |
| *Oscillospira* | 0.0508 |  | *Treponema* | 0.0762 |  | *Prevotella* | 0.0980 |
| *Faecalibacterium* | 0.0432 |  | *Desulfovibrio* | 0.0477 |  | *Oscillospira* | 0.0483 |
| *CF231* | 0.0349 |  | *Oscillospira* | 0.0335 |  | *Ruminococcus* | 0.0240 |
| *Campylobacter* | 0.0294 |  | *Parabacteroides* | 0.0250 |  | *Parabacteroides* | 0.0218 |
| *Parabacteroides* | 0.0285 |  | *Ruminococcus* | 0.0228 |  | *CF231* | 0.0182 |
| *Bacteroides* | 0.0158 |  | *YRC22* | 0.0182 |  | *Desulfovibrio* | 0.0154 |
| *Ruminococcus* | 0.0130 |  | *CF231* | 0.0175 |  | *Akkermansia* | 0.0143 |
|  |  |  | *Akkermansia* | 0.0149 |  | *Faecalibacterium* | 0.0111 |
|  |  |  |  |  |  | *YRC22* | 0.0105 |

Supplementary Figure S1. Heatmaps of relative abundance of bacterial OTUs. The classifications included phylum (a), class (b), order (c), family (d), genus (e), species (f).


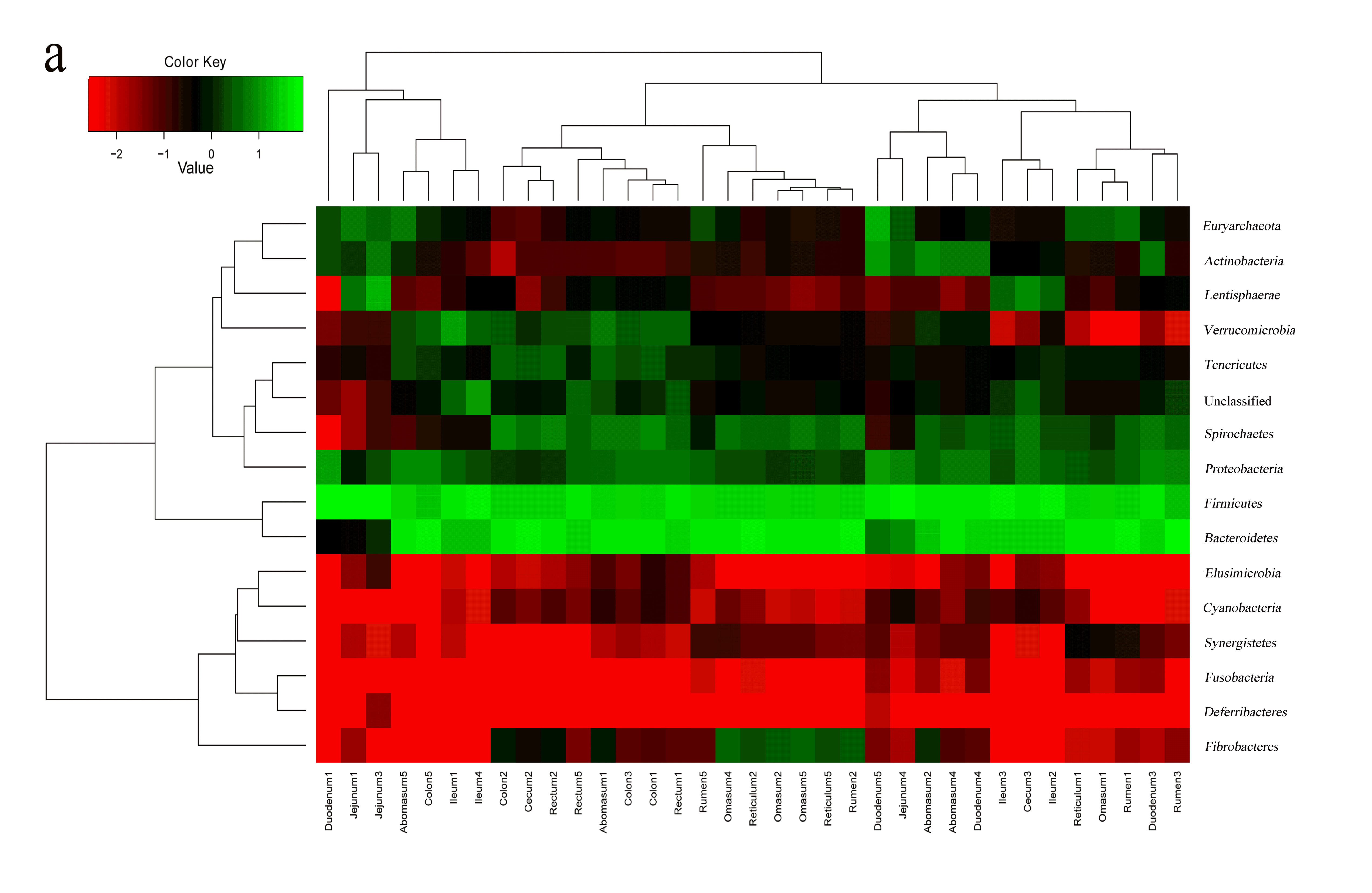


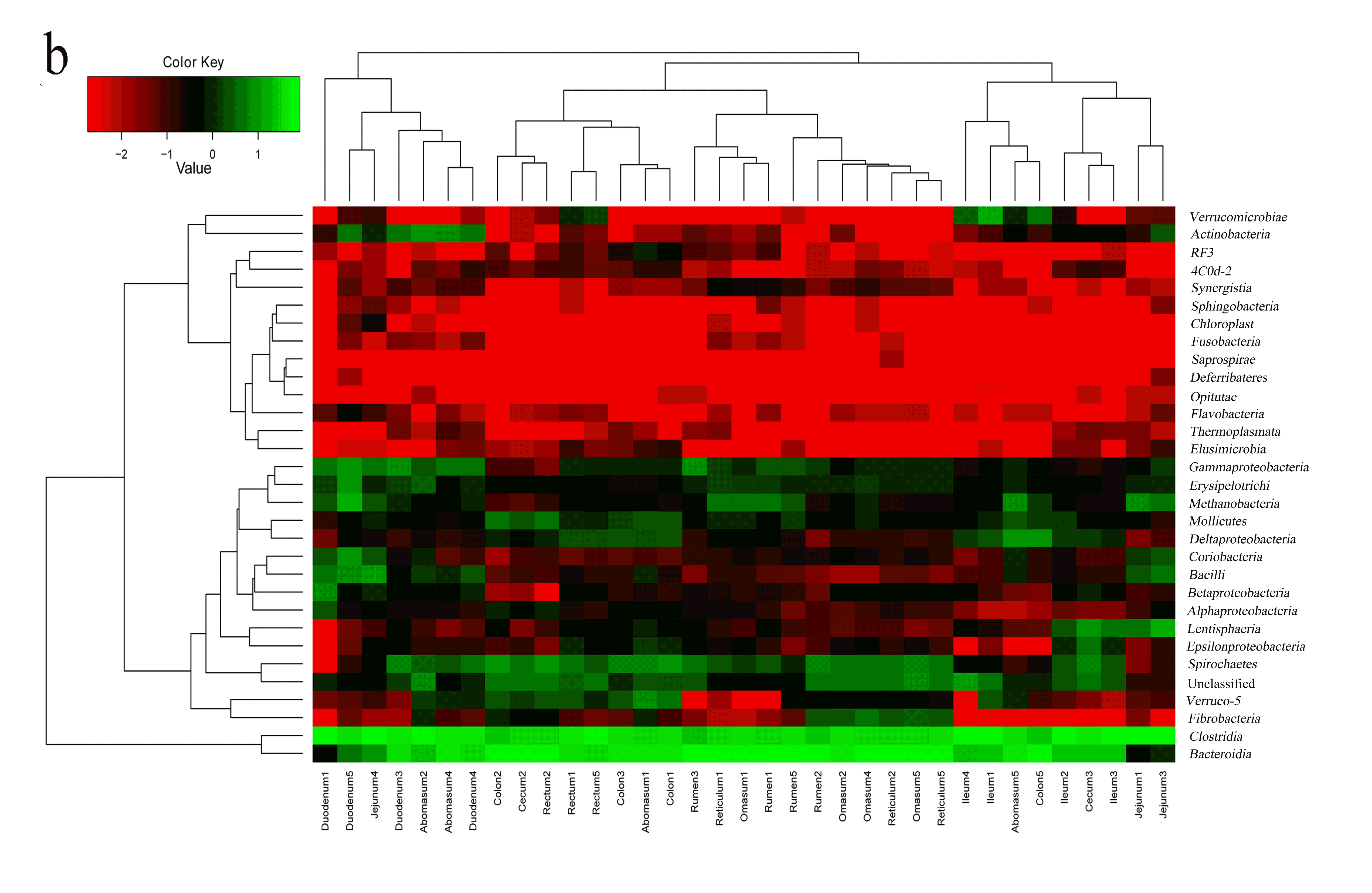


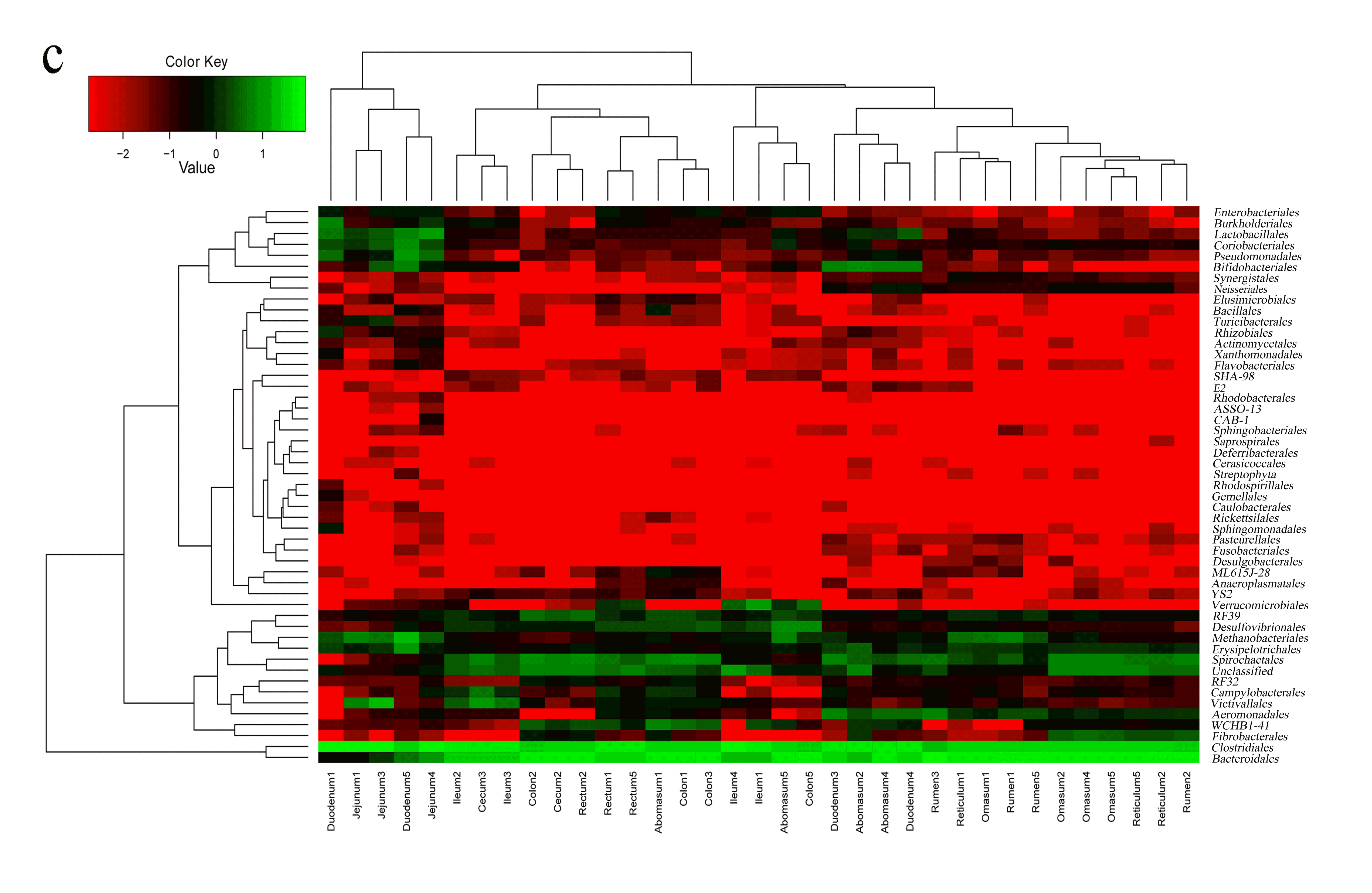


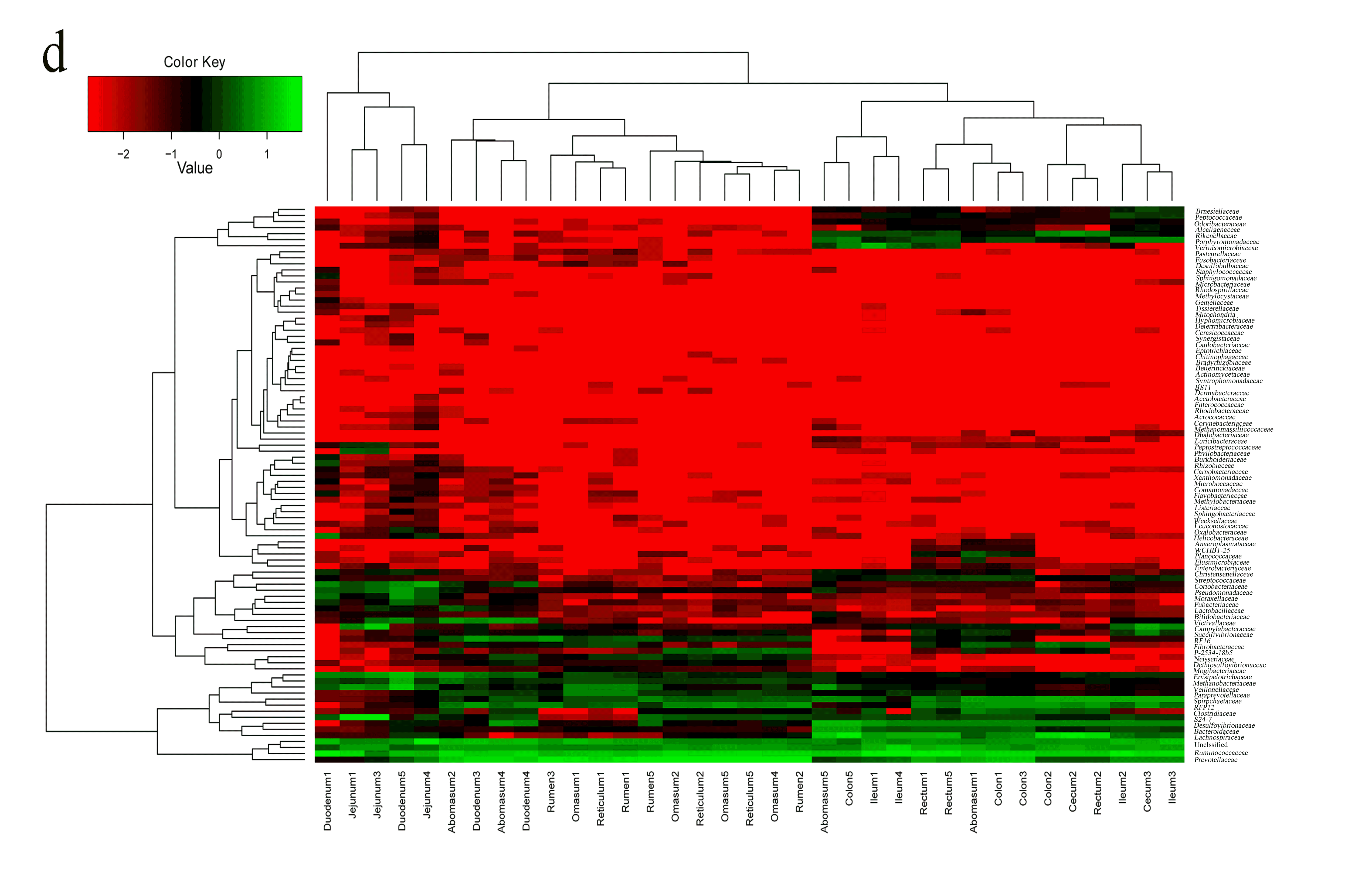


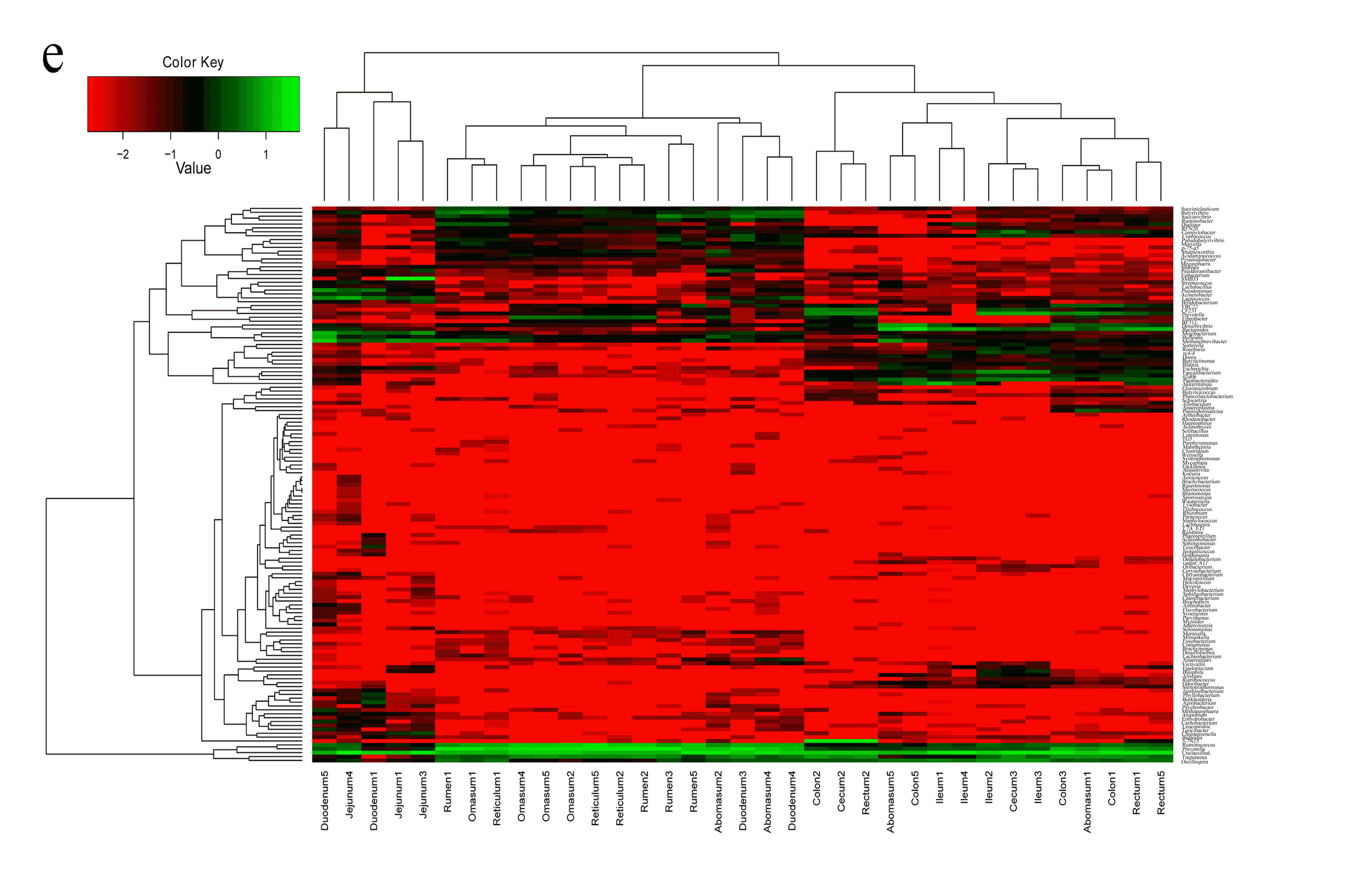


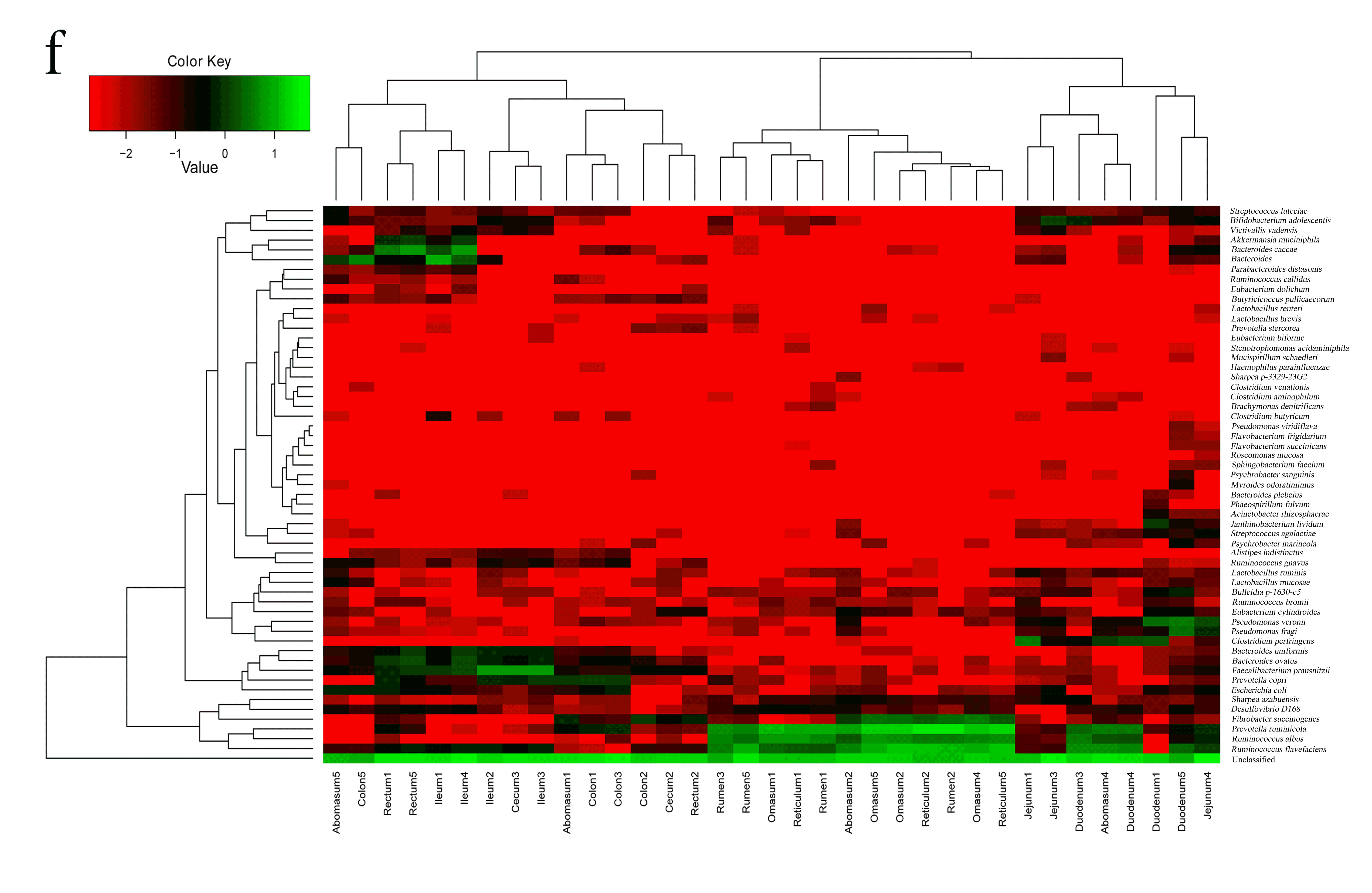


Supplementary Figure S2. Venn diagram of the unique and shared bacterial genera (with the percentage of >1% colonized in segment) at the genus level in the sheep GIT. Venn diagrams depictions of samples from rumen, reticulum, omasum, and abomasum (a). Venn diagrams depictions of samples from duodenum, jejunum, and ileum(b). Venn diagrams depictions of samples from cecum, colon, and rectum (c).


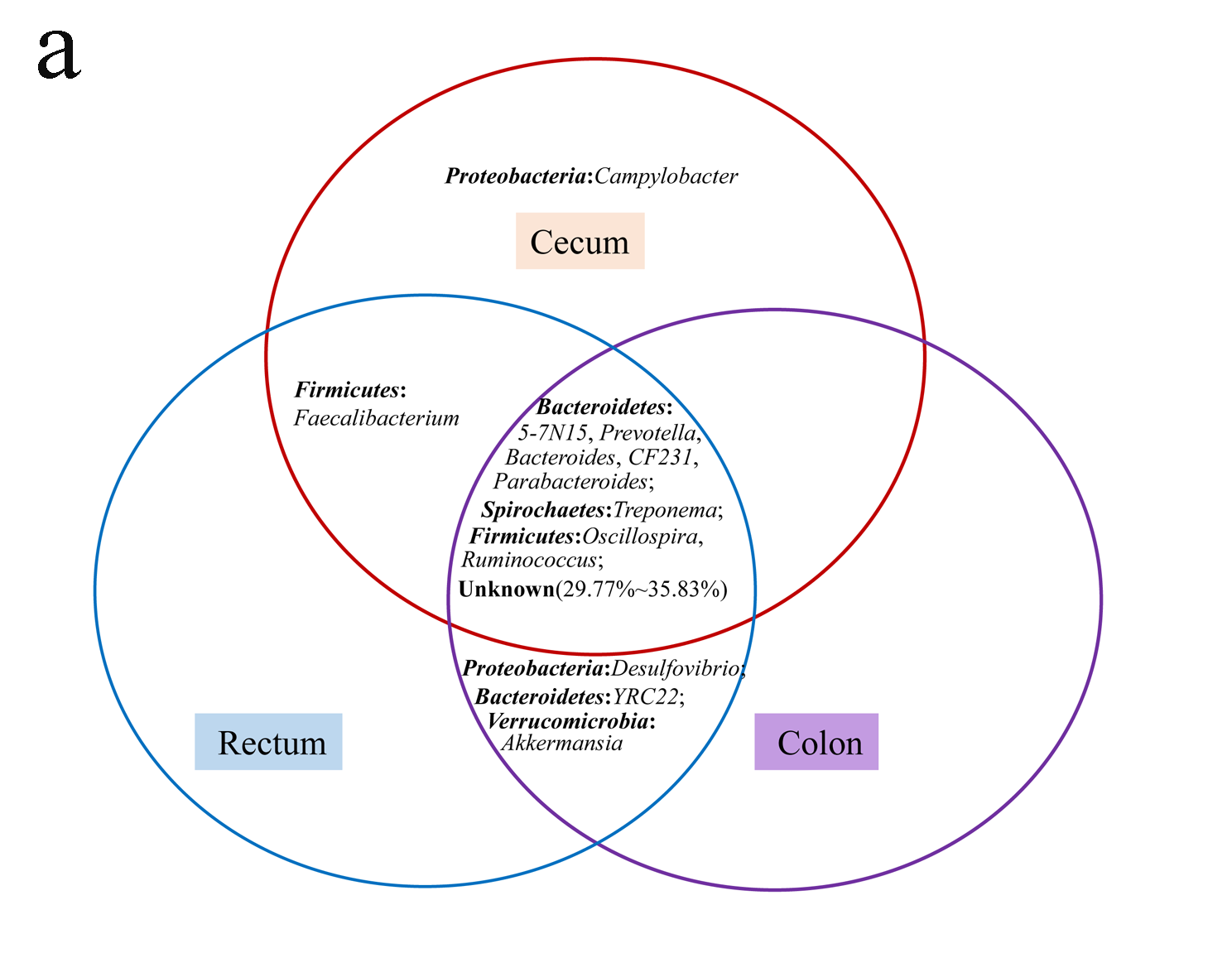


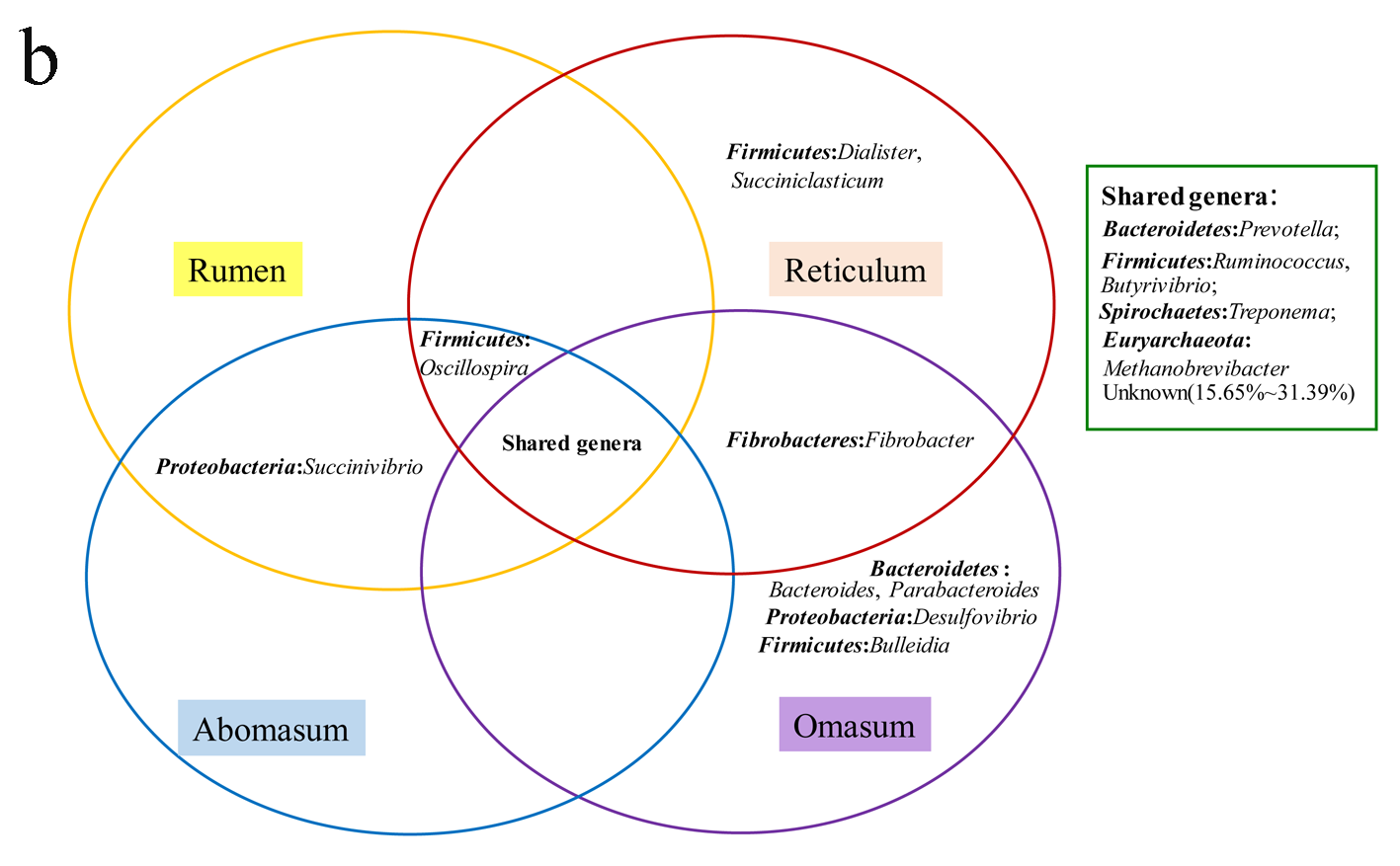


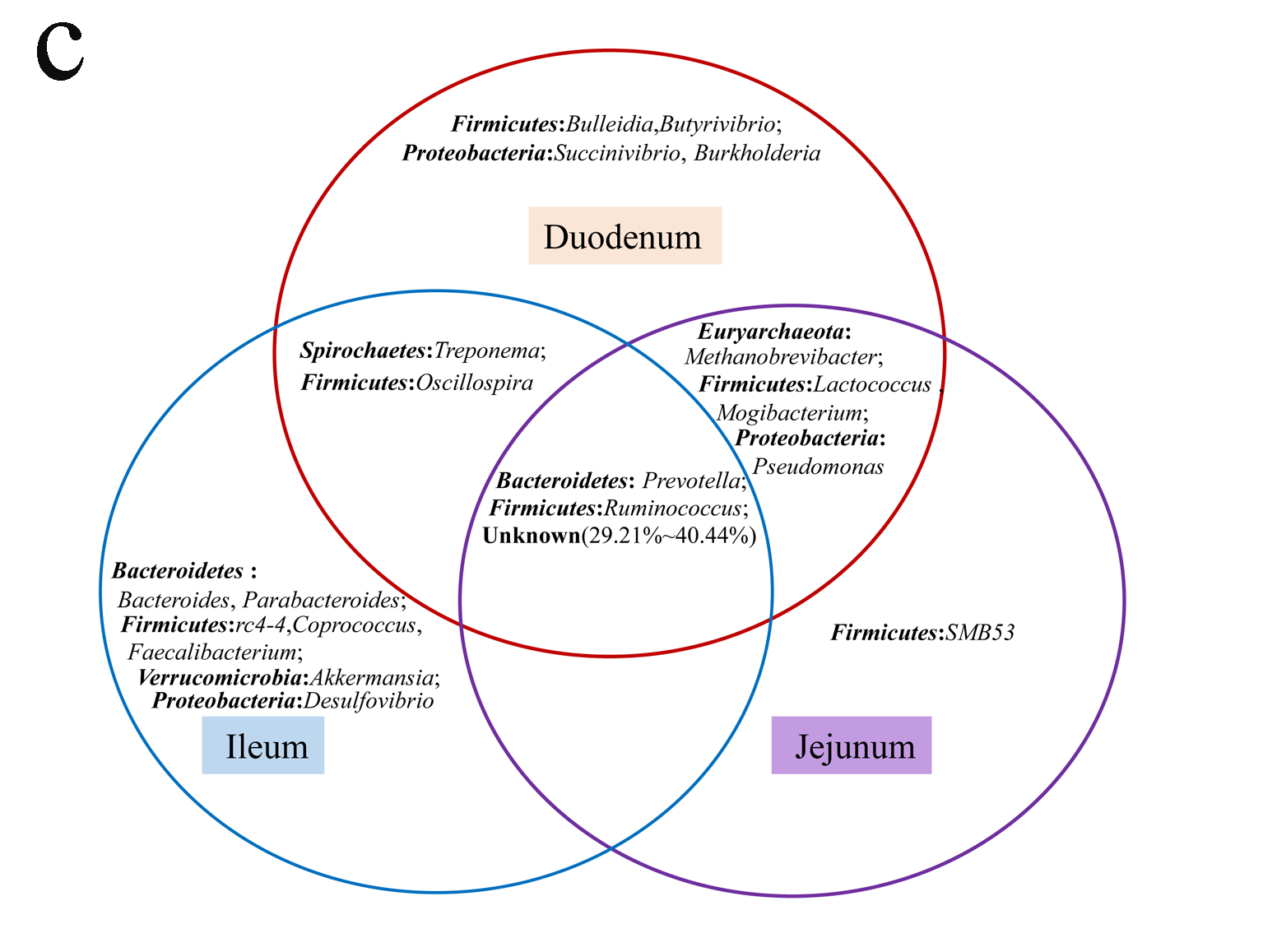

Supplement: Supplementary file 1 — Additional file 1. Additional tables and figures. [file 13568_2017_378_MOESM1_ESM.doc]
